# Repression-free utrophin-A 5’UTR variants

**DOI:** 10.22099/mbrc.2019.34240.1421

**Published:** 2019-09

**Authors:** Debasish Malik, Utpal Basu

**Affiliations:** Department of Molecular Biology and Biotechnology, University of Kalyani, Kalyani, Nadia, 741235 India

**Keywords:** DMD, Utrophin, 5′RACE

## Abstract

Mutation in the dystrophin gene results Duchenne Muscular Dystrophy (DMD), an X-linked fatal neuromuscular disorder. Dystrophin deficiency can be compensated by upregulation of utrophin, an autosomal homologue of dystrophin. But the expression of utrophin in adults is restricted to myotendinous and neuromuscular junctions. Therefore utrophin upregulation throughout the muscle fiber can only be achieved if we understand regulatory mechanisms behind its expression. Utrophin-A 5′UTR mediated repression of translation was reported earlier. In this article, we present evidences of two transcript variants of utrophin-A that do not confer repression to the downstream reporter ORF in mouse myoblast C2C12 cells. These repression-free variants may be targeted for utrophin upregulation.

## INTRODUCTION

Pathology of Duchenne Muscular Dystrophy (DMD) arises from the mutation in dystrophin gene [1]. Upregulation of utrophin, an autosomal homologue of dystrophin has been considered as a promising approach for DMD therapy [2-5]. Although many utrophin-isoforms exist, in muscle utrophin-A is expressed [6]. Therefore, understanding the regulatory mechanisms of utrophin-A expression would help development of therapeutic strategies for its upregulation in DMD patients. But utrophin-A mRNA is repressed at the level of translation. MicroRNA targeting its 3′UTR and sequence elements at its 5′UTR have been attributed to this repression [7, 8]. The promoter driving utrophin-A expression is TATA less and Transcription Start Site (TSS) is not well defined. However, we hypothesized that because of the presence of many TSS, all utrophin-A 5′UTR variants may not be repressive in terms of translation. We performed 5′ RACE and identified two novels TSS that generate repression-free 5′UTR variants of utrophin-A. Upregulation of these utrophin-A 5′UTR variants can be targeted to achieve utrophin upregulation.

## MATERIALS AND METHODS

**5**′**RACE:** Total RNA was isolated from mouse myoblast C2C12 cells with TriZol (Invitrogen), DNase I (NEB) treated and purified with phenol-chloroform. After alcohol precipitation and washing with 70% ethanol RNA was treated with CIAP (NEB) and purified with phenol-chloroform. The dephosphorylated RNA was treated with Tobacco Acid Pyrophosphates (TAP) (Epicentre) followed by purification. 10 µg RNA was ligated with 7 µg RNA oligo (5′ATT AAT ACG ACT CAC TAT AGG GAG ACC CAA GCT GGC TAG CGT TTA AAC TTA AGC TTG GTA CCA TGT CCG TCC TGA CGC CGC TGC TGC TGC GGG GCT TGA CAG GCT CGG CCC GGC GGC TCC CAG TGC CGC GCG CCA AGA TCC ATT CGT TGG GGG ATC C3′) by T4 RNA ligase (Sibenzyme) at 16°C for overnight. 

cDNA was synthesized after ligation with MMLV reverse transcriptase (Epicentre) with utrophin specific reverse primer (Table 1) according to the manufacturer’s protocol, at 37°C for 45 min, 30 min at 45°C and 10 min at 50°C. RACE products were obtained with nested PCR using gene and RNA oligo specific primers (Table 1), cloned and sequenced. CIAP treated but TAP untreated RNA was used as control.

**Cloning:** A and A´are two splice variants of utrophin transcript produced from the utrophin-A promoter. The utrophin (Utrn) variants were named according to TSS relative to the start codon. The numbers after Utrn A and A´ represent distance of different TSS from start codon in nucleotide. Four different utrophin-A 5′UTRs (Utrn A394, Utrn A267, Utrn A207 and Utrn A´157) were cloned in empty pGL3 control vector at NcoI site followed by correction of ‘CC’ within NcoI site with primer pairs (Table 1) and using QuikChange Kit (Stratagene). 

**Table 1:** Primers used in the study

**Primer Name**	** Primer sequence**	**Purpose**
T7 UtrnA507 Fw	5′ACTGCTAATACGACTCACTATAGGGTTGTGGAGTCGCCCTTC3′	Used for in vitro transcription
T7UtrnA394 Fw	5′GATTATATAATACGACTCACTATAGGGGGGGAGCGGCGCCCCTTTTC3′	
T7UtrnA267 Fw	5′GATTATATAATACGACTCACTATAGGGTCAGGGGGCGTTTCCAATCG3′	
T7UtrnA207 Fw	5′GATTATATAATACGACTCACTATAGGG GGATCTTGTCGGGCTTTCCACG3′	
T7UtrnA′ 157 Fw	5′GATTATATAATACGACTCACTATAGGG TTGGTGCGCAGTCCCGC3′	
T7pRLTK Fw	5′GAGTACTTAATACGACTCACTATAGGCTAGCCACCA3′	
pGL3T_50_ Rev	5′ T_50_CTCTAGAATTACACGGCGATCTTTCCG3′	
pRLTKT_50_ Rev	5′ T_50_CCGCTCTAGAATTATTGTTCATTTTTGAGAACTC3′	
Utrophin specific Reverse	5′GTCATCTTGCTCCTGGAACGTGTC3′	Used in 5′RACE
Gene specific primer1	5′GATGTTCCTGTGAGGCCTTCGAGAA3′	
Gene specific primer 2	5′TCATCAGGCCTGGCTTCAAGGTC3′	
RNA oligo specific primer 1	5′ATGTCCGTCCTGACGCCGC3′	
RNA oligo specific primer 2	5′CGCGCGCCAAGATCCATTC3′	
Del Fw	5′TCATTCAAGATGGAAGACGCCAAAAACATAAAGAAAGGC3′	Used in “CC” deletion mutation
Del Rev	5′GTCTTCCATCTTGAATGAGTTTCAGTATAATCCAAAGGCTTTC3′	
Utrn394Fw	5′GCTAGCGTTTGGACTGTTTTGTT3′	
Utrn394Rev	5′CCCCTGACTTGTGCAATCTTC3′	for Real Time PCR
Utrn207Fw	5′GGGCTTTCCACGTTTCACTTAA3′	
Utrn207Rev	5′GGTTCCTGGGCGTGTTC3′	
UtrnFw	5′GATGACCTTGCTGAATGCAAGG3′	
UtrnRev	5′GAGCTGCTGCAGCTGTTTCTTC3′	

**In vitro transcription:** T7 RNA polymerase promoter containing forward primers (Table 1) were designed corresponding to four different 5′RACE products of 5′UTRs of utrophin-A (and A´, transcript variant of utrophin-A), 507nt utrophin-A 5′UTR fragment that is known to represses translation of downstream ORF [7-9] and renilla luciferase of pRLTK respectively. T_50_ tailed reverse primers (Table 1) were specific for firefly and renilla luciferase. Template for in vitro synthesis of RNA was prepared with PCR using proper template and primers. T7 RNA polymerase (NEB), m^7^G(5′)ppp(5′)G cap analog (NEB), NTPs were used in vitro transcription with proper template according to the manufacturer’s protocol followed by DNase I (NEB) treatment. 

**Transfection and Luciferase assay:** 50,000 cells were seeded per well in 24 well plate 24 hours before transfection. 100 ng firefly luciferase reporter mRNA and equimolar amount of capped, A_50_ tailed reporter mRNAs were transfected with Lipofectamine 2000 (Invitrogen) along with 1 ng renilla luciferase reporter mRNA in each well. Luciferase assay was done four hours after transfection using DualGlo Luciferase Assay Kit (Promega).

**Quantitative PCR:** Absolute quantification of utrophin-A was determined using iTaq Universal SYBR Green (Bio-Rad) with different primer pairs (Table 1). Standard curve was prepared by serial dilution of PCR amplified templates.

## RESULTS AND DISCUSSION

**Figure 1:**
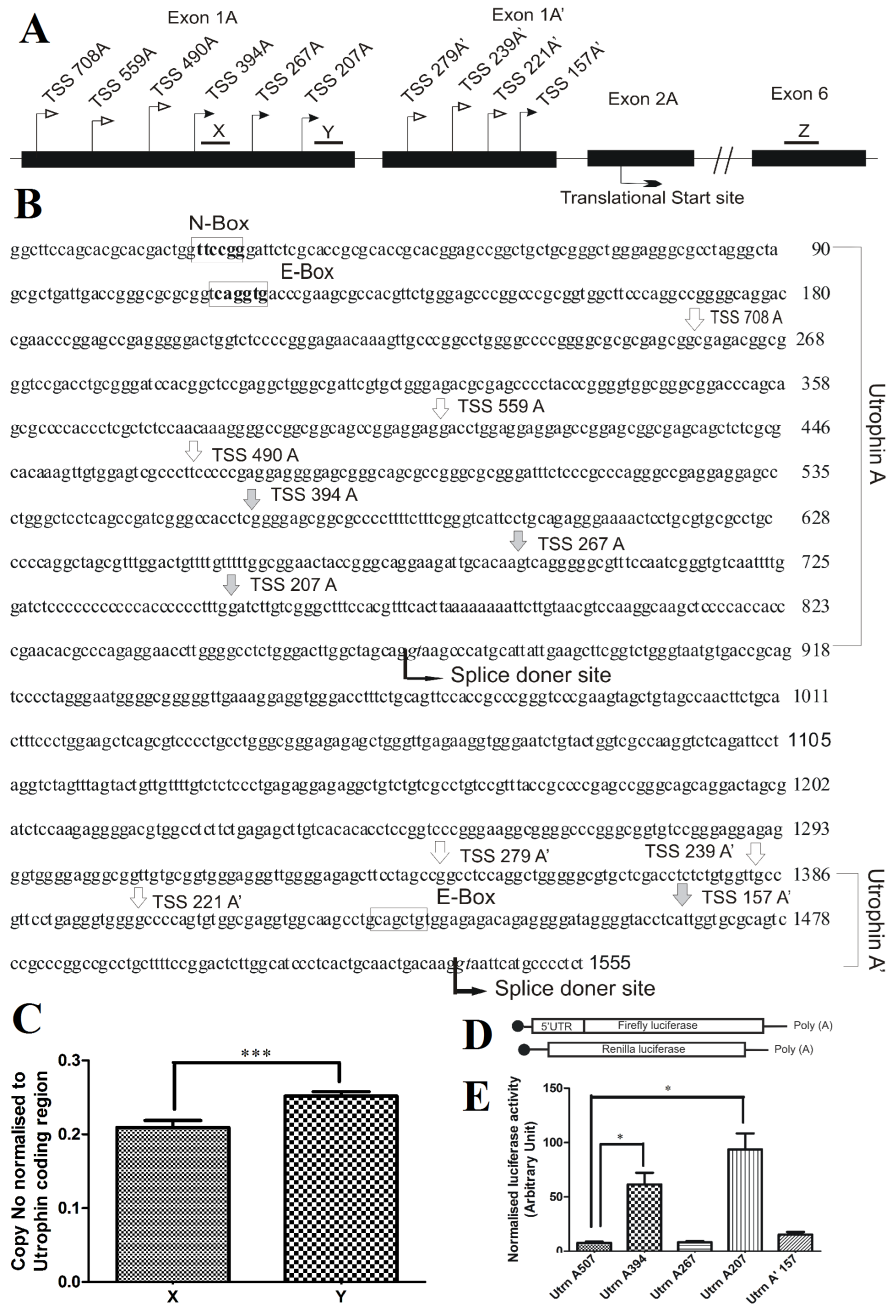
Transcription Start Sites in utrophin A genomic region. (A) Schematic presentation of TSS. A and A´ are two splice variants of utrophin transcript produced from the utrophin-A promoter. The numbers after TSS represent distance of different TSS in nucleotide from start codon. (B) Positions of TSS in genomic sequence. The white arrow indicates the previously reported TSS and the grey arrow indicates the new TSS of utrophin A and A′. (C) Existence of TSS downstream of already reported TSS 490A. Utrophin-A mRNA copy number was determined with qPCR using different primer pairs. Amplicon positions are shown by black lines (X, Y, Z) in Figure 1 (A). Copy number normalized against utrophin-A coding region was plotted as mean ± SD (n=6 independent experiments). Student’s t test was used to analyze the data. The asterisk denotes P<0.0001. Difference present between copy number corresponding to amplicon X and Y confirmed subsistence of TSS between denoted amplicons. Z represents utrophin coding region amplicon used for normalisation. (D) Schematic presentation of reporter mRNAs. The utrophin-A 5′UTRs found in the 5′RACE experiment was placed at the upstream of firefly luciferase ORF in pGL3. In vitro transcribed capped, A_50_ tailed reporter mRNA with 5′UTRs were transfected in C2C12 cells followed by estimation of reporter activity. Renilla reporter mRNA was used as transfection control. (E) Regulatory roles of different utrophin-A 5′UTRs. Two repression-free variants were identified. Results presented as mean ±SD (n=6 independent experiments). Student’s t test was done to analyze the data. The asterisk denotes the statistically significant (P< 0.0001) difference

From mouse myoblast C2C12 cells, with RNA ligase mediated 5′RACE we have identified three products corresponding to three novel transcription start sites that have not been reported earlier. We have also detected one novel TSS of utrophin-A′, a splice-variant of utrophin-A that was previously reported (Fig. 1A, B) [6]. In order to confirm the existence of novel TSS, we performed absolute quantification of utrophin-A transcripts with two primer pairs downstream of reported TSS 490A (Fig. 1A). The difference in copy number validates the existence of novel TSS (Fig. 1C).

The presence of many transcription start sites (TSS) at utrophin-A promoter indicates the existence of variation in 5'UTR of utrophin-A mRNA. We have previously reported that utrophin-A transcript is translationally repressed and the repression is mediated through its 5′ and 3′UTRs. 5′UTR mediated repression of translation has been attributed to two sequence elements and one upstream ORF present in 507 nt long utrophin-A 5′UTR fragment. Having identified three novel TSS and three new 5′UTR variants, we asked if any of the variants posses comparatively less repressor activity with respect to translation of downstream coding region. We therefore made reporter constructs with firefly luciferase ORF having utrophin-A 5′UTR variants at its upstream. In vitro transcription of these constructs made m^7^G capped, poly A tailed (A_50_) firefly luciferase reporter mRNAs with different 5′UTR variants of utrophin-A. Transfection of reporters having length of 394 and 207 nt utrophin-A 5′UTRs in C2C12 cells showed much higher reporter activity compared to well studied 507 nt long 5′UTR fragment of utrophin-A transcript.

The present study, therefore, identified novel utrophin-A 5′UTR variants namely Utrn A394 and Utrn A207 from C2C12 cells which are repression-free. These variants or transcription from these TSS would therefore be targeted to achieve utrpophin-A upregulation.
